# Supplementary material for: Cytosine and adenine deaminase base-editors induce broad and nonspecific changes in gene expression and splicing
Source: Commun Biol. 2021 Jul 16;4:882. doi: 10.1038/s42003-021-02406-5 (PMC8285404; doi:10.1038/s42003-021-02406-5)
Supplement: Supplementary file 2 — Supplementary Information [file 42003_2021_2406_MOESM2_ESM.pdf]

Supplementary Information for

Cytosine and adenine deaminase base-editors induce broad and nonspecific changes in gene expression and splicing

Supplementary Methods

Identification of off-target RNA SNVs

Raw data were initially inspected using FastQC (v0.11.8) and reads were trimmed with Trim Galore (v0.6.1) using default parameters for pair-end data to remove both low-quality reads and adaptors. Then, high quality reads were aligned onto the GRCh38 human transcriptome with STAR (v2.5.0a). After the preprocessing, RNA-seq reads were passed to the REDItoolDenovo.py script from REDItools package using parameters "-t 10 -e -d -l -p -u -m20 -T6-0 -W -v 1 -n 0.0" to identify RNA SNVs candidates. At the end of the run, all variants were further filters to removed variant sites identified in GFP-alone to find the base editor-induced RNA SNVs.

Supplementary Figures

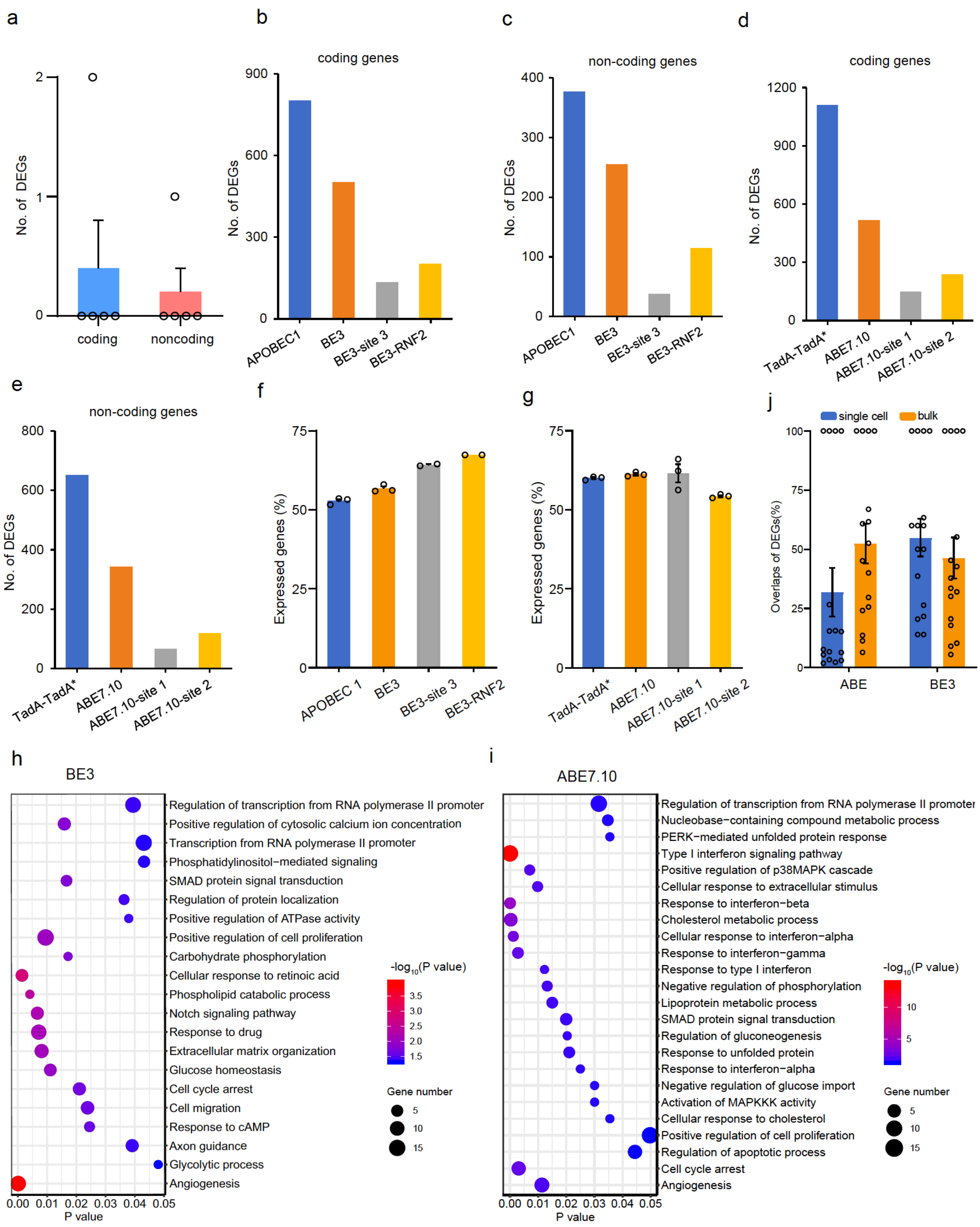

Supplementary Figure 1. Identification and characterization of DEGs induced by DNA base editors.

a) We randomly selected three repeats from six repeats of GFP cells as control groups and the other three repeats as treatment groups. Bar plots showing the number of differentially expressed coding and noncoding genes compared GFP-transfected cells with GFP groups.

b) Bar plots showing the number of differentially expressed coding genes identified in HEK293T cells transfected with APOBEC1, BE3, BE3-site 3 and BE3-RNF2. GFP group serves as the control for all comparisons.

c) Bar plots showing the number of differentially expressed noncoding genes identified in HEK293T cells transfected with APOBEC1, BE3, BE3-site 3 and BE3-RNF2. GFP group serves as the control for all comparisons.

d) Bar plots showing the number of differentially expressed coding genes identified in HEK293T cells transfected with TadA-TadA*, ABE7.10, ABE7.10-site 1 and ABE7.10-site 2. GFP group serves as the control for all comparisons.

e) Bar plots showing the number of differentially expressed noncoding genes identified in HEK293T cells transfected with TadA-TadA*, ABE7.10, ABE7.10-site 1 and ABE7.10-site 2. GFP group serves as the control for all comparisons. Supplementary Figure 1a-e were corresponding to Supplementary Data 1.

f, **g**) The ratio of the expressed genes (FPKM > 1) in DEGs induced by BE editors. Error bars represent S.D. for 3 independent experiments.

h, **i**) Gene Ontology enrichment analysis for biological process of DEGs identified in BE3 or ABE7.10 groups compared with GFP samples. Corresponding data were presented in Supplementary Data 2

j) The ratio of shared DEGs between any two samples in single-cell RNA-seq samples of BE3-site3 and ABE7.10-site1 groups and the ratio of shared DEGs between any two samples in bulk RNA-seq samples. Error bars represent S.D. for 12 independent experiments.

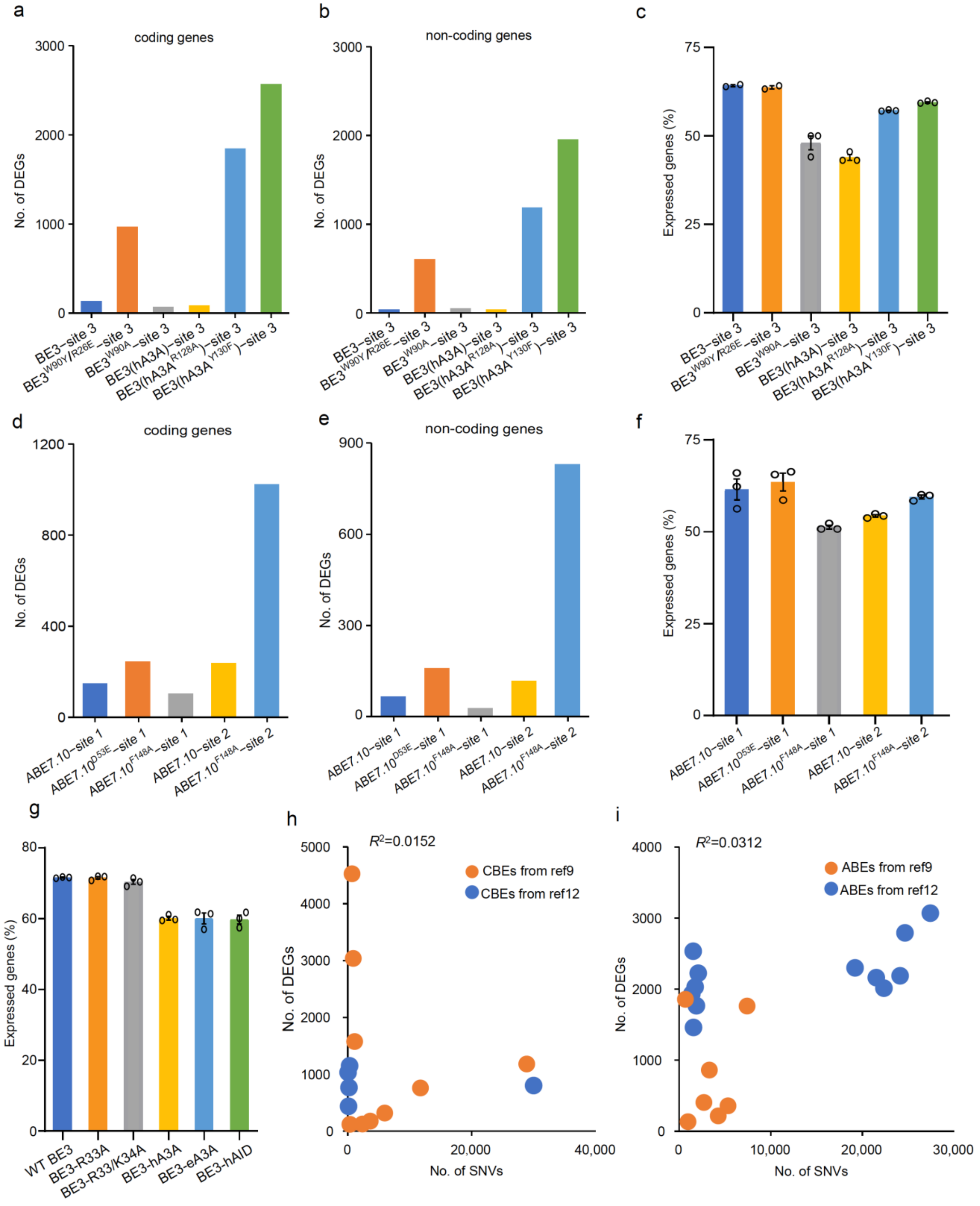

Supplementary Figure 2. Identification of DEGs induced by engineered BE variants editors.

a) Bar plots showing the number of differentially expressed coding genes identified in HEK293T cells transfected with BE3-site 3, BE3$^{W90Y/R126E}$-site 3, BE3^{W90A}-site 3, BE3(hA3A)-site 3,

BE3(hA3A^{R128A})-site 3, and BE3(hA3A^{Y130F})-site 3 groups. GFP group serves as the control for all comparisons.

b) Bar plots showing the number of differentially expressed noncoding genes identified in HEK293T cells transfected with BE3-site 3, BE3$^{W90Y/R126E}$-site 3, BE3^{W90A}-site 3, BE3(hA3A)-site 3, BE3(hA3A^{R128A})-site 3, and BE3(hA3A^{Y130F})-site 3 groups. GFP group serves as the control for all comparisons.

c) The ratio of the expressed genes (FPKM > 1) in DEGs induced by BE3-site 3, BE3$^{W90Y/R126E}$-site 3, BE3^{W90A}-site 3, BE3(hA3A)-site 3, BE3(hA3A^{R128A})-site 3, and BE3(hA3A^{Y130F})-site 3.

d) Bar plots showing the number of differentially expressed coding genes identified in HEK293T cells transfected with ABE7.10-site 1, ABE7.10^{D53E}-site 1, ABE7.10^{F148A}-site 1, ABE7.10-site 2 and ABE7.10^{F148A}-site 2 groups. GFP group serves as the control for all comparisons.

e) Bar plots showing the number of differentially expressed noncoding genes identified in HEK293T cells transfected with ABE7.10-site 1, ABE7.10^{D53E}-site 1, ABE7.10^{F148A}-site 1, ABE7.10-site 2 and ABE7.10^{F148A}-site 2 groups. GFP group serves as the control for all comparisons.

f) The ratio of the expressed genes (FPKM > 1) in DEGs induced by ABE7.10-site 1, ABE7.10^{D53E}-site 1, ABE7.10^{F148A}-site 1, ABE7.10-site 2 and ABE7.10^{F148A}-site 2.

g) The ratio of the expressed genes (FPKM > 1) in DEGs induced by WT BE3, SECURE-BE3(R33A), SECURE-BE3(R33A/K34A), hA3A-BE3, eA3A-BE3, and hAID-BE3.

h) Correlations between numbers of DEGs and numbers of off-target RNA SNVs in CBEs-treated groups. R^2 values were calculated by robust linear regressions on numbers of DEGs and numbers of off-target RNA SNVs.

i) Correlations between numbers of DEGs and numbers of off-target RNA SNVs in ABEs-treated groups. R^2 values were calculated by robust linear regressions on numbers of DEGs and numbers of off-target RNA SNVs. Error bars represents S.D. for 3 independent experiments.

Supplementary Figure 2a,b,d were corresponding to Supplementary Data 3.

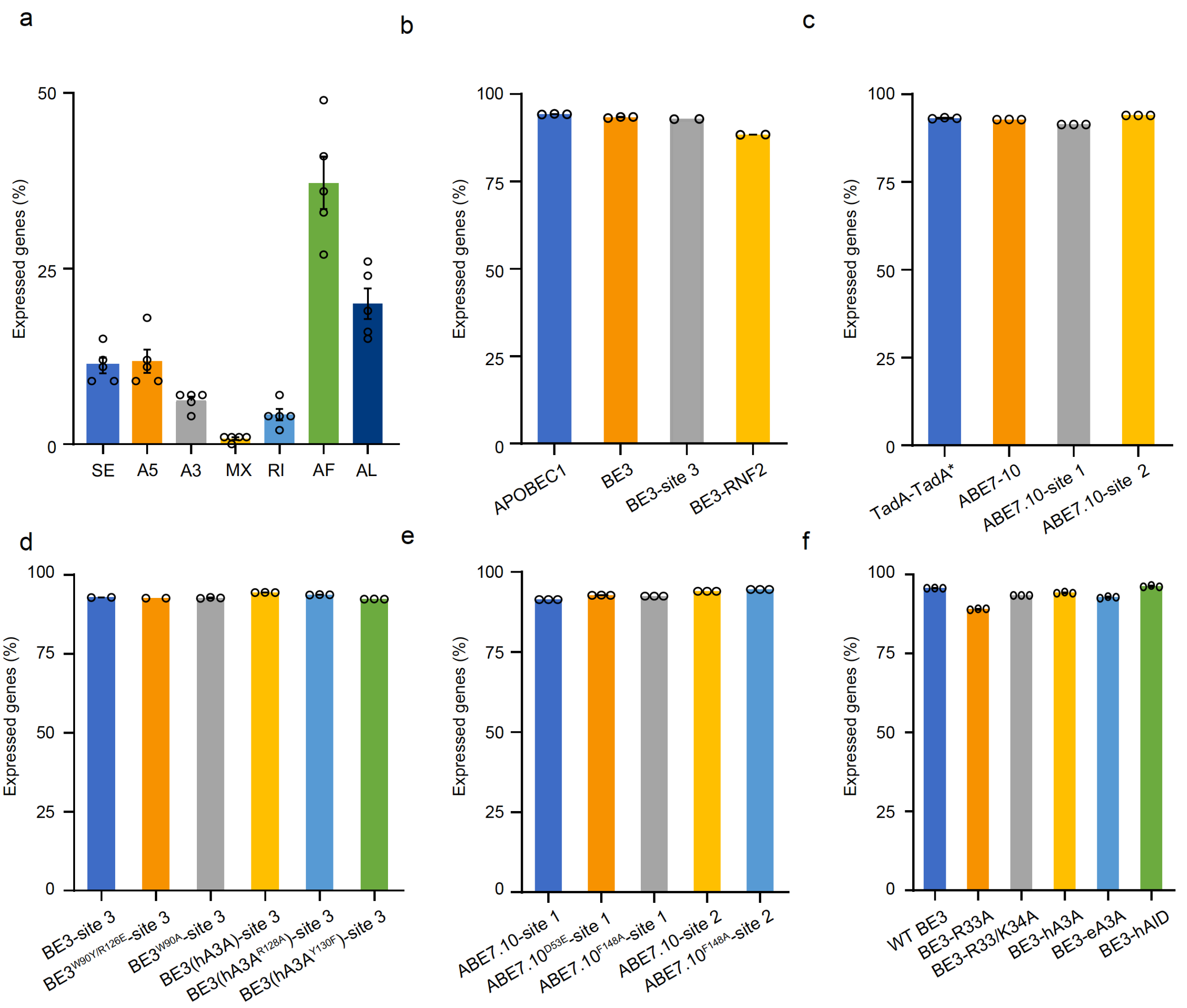

Supplementary Figure 3. Identification and characterization of DAS events induced by DNA base editors.

a) We randomly selected three repeats from six repeats of GFP cells as control groups and the other three repeats as treatment groups. Bar plots showing the number of DAS events compared GFP-transfected cells with GFP groups.

b) The ratio of the expressed genes (FPKM > 1) in DAS events related genes induced by APOBEC1, BE3, BE3-site 3 and BE3-RNF2.

c) The ratio of the expressed genes (FPKM > 1) in DAS events related genes induced by TadA-TadA*, ABE7.10, ABE7.10-site 1 and ABE7.10-site 2.

d) The ratio of the expressed genes (FPKM > 1) in DAS events related genes induced by BE3-site 3,

BE3$^{W90Y/R126E}$-site 3,BE3^{W90A}-site 3, BE3(hA3A)-site 3, BE3(hA3A^{R128A})-site 3,and BE3(hA3A^{Y130F})-site 3.

e) The ratio of the expressed genes (FPKM > 1) in DAS events related genes induced by ABE7.10-site 1,ABE7.10^{D53E}-site 1, ABE7.10^{F148A}-site 1, ABE7.10-site 2 and ABE7.10^{F148A}-site 2 groups.

f) The ratio of the expressed genes (FPKM > 1) in DAS events related genes induced by WT BE3, SECURE-BE3(R33A), SECURE-BE3(R33A/K34A), hA3A-BE3, eA3A-BE3, and hAID-BE3. Error bars represent S.D. for 3 to 5 independent experiments.

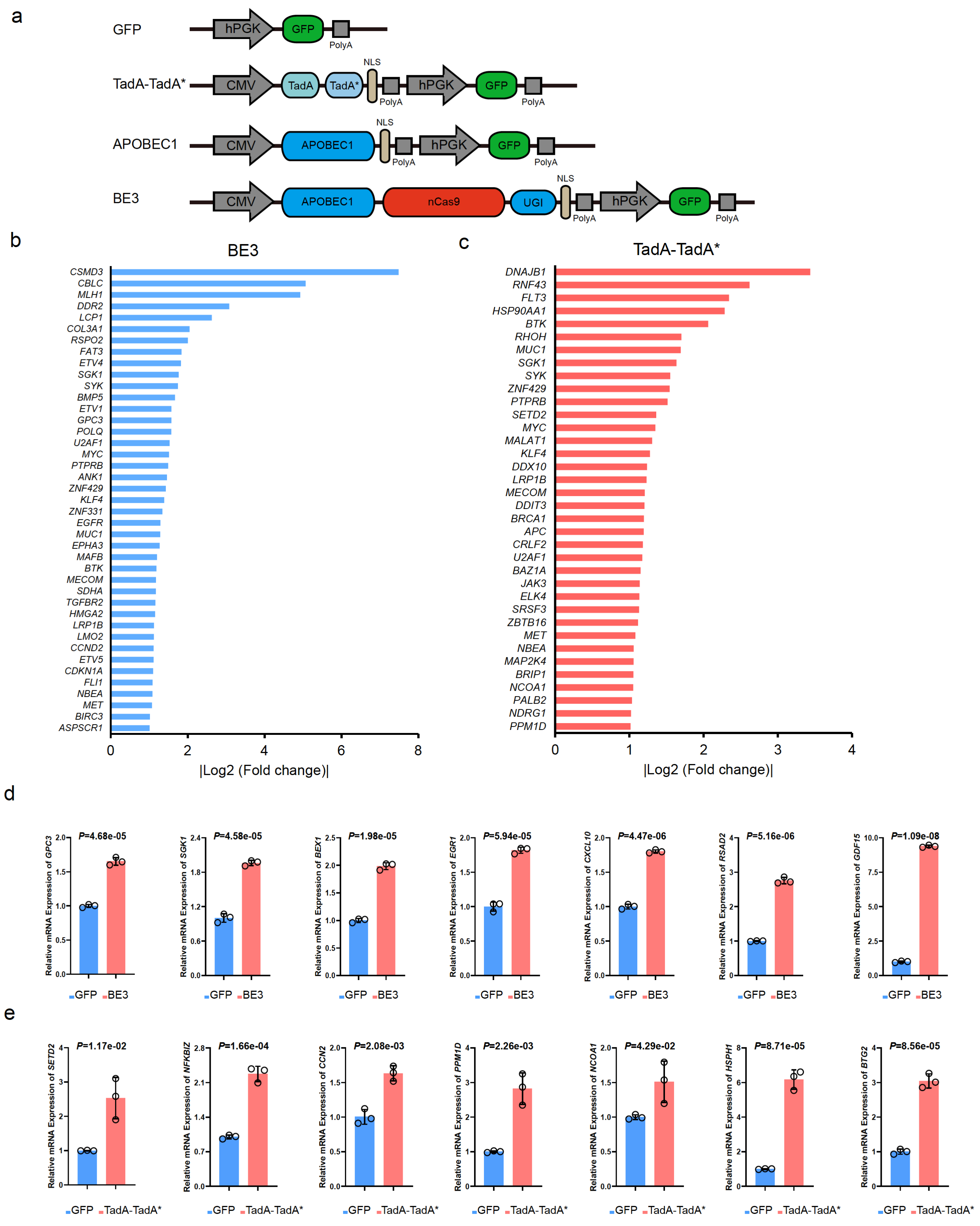

Supplementary Figure 4. Validation of DEGs identified from RNA-seq data of HEK293T cells.

a) Schematics of plasmids. The schematics show the plasmids used in this study.

b) Cancer-related genes in BE3-induced DEGs.

c) Cancer-related genes in TadA-TadA*-induced DEGs.

d) Validation of DEGs in BE3-treated groups by qPCR. The tested genes were randomly selected and the RNA levels are shown relative to those of the control GFP groups and were normalized to 1. Data of qPCR are presented as the mean ± s.e.m. from three independent.

e) Validation of DEGs in TadA-TadA*-treated groups by qPCR. The tested genes were randomly selected and the RNA levels are shown relative to those of the control GFP groups and were normalized to 1. Data of qPCR are presented as the mean ± s.e.m. from three independent. Error bars represent S.D. for 3 independent experiments.

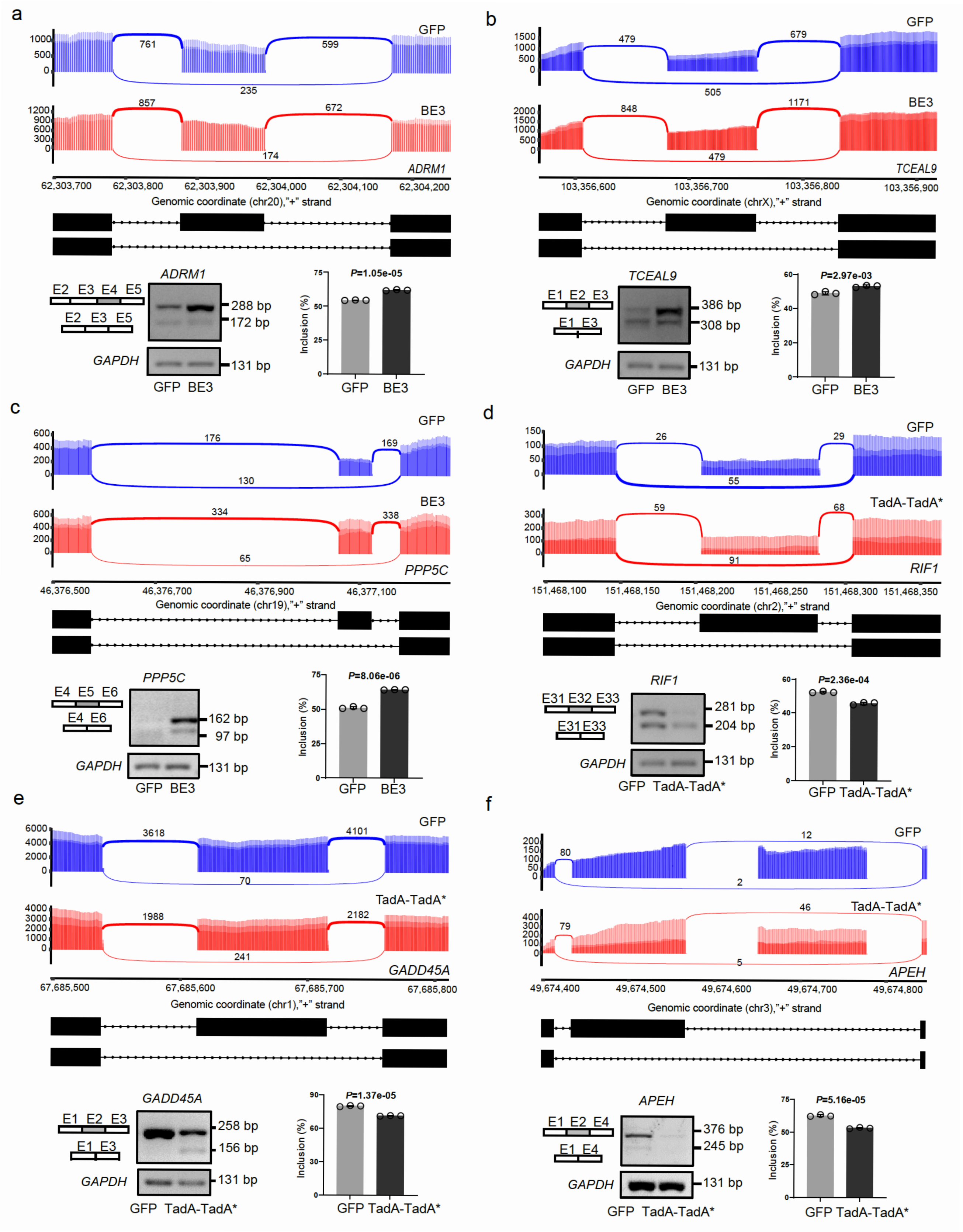
a
GFP
761
599
235
BE3
857
672
174
ADRM1
62,303,700
62,303,800
62,303,900
62,304,000
62,304,100
62,304,200
Genomic coordinate (chr20),"+" strand
E2 E3 E4 E5
E2 E3 E5
ADRM1
288 bp
172 bp
GAPDH
131 bp
GFP BE3
P=1.05e-05
Inclusion (%)
GFP BE3
b
GFP
479
679
505
BE3
848
1171
479
TCEAL9
103,356,600
103,356,700
103,356,800
103,356,900
Genomic coordinate (chrX),"+" strand
E1 E2 E3
E1 E3
TCEAL9
386 bp
308 bp
GAPDH
131 bp
GFP BE3
P=2.97e-03
Inclusion (%)
GFP BE3
c
GFP
176
169
130
BE3
334
338
65
PPP5C
46,376,500
46,376,700
46,376,900
46,377,100
Genomic coordinate (chr19),"+" strand
E4 E5 E6
E4 E6
PPP5C
162 bp
97 bp
GAPDH
131 bp
GFP BE3
P=8.06e-06
Inclusion (%)
GFP BE3
d
GFP
26
29
55
TadA-TadA*
59
68
91
RIF1
151,468,100
151,468,150
151,468,200
151,468,250
151,468,300
151,468,350
Genomic coordinate (chr2),"+" strand
E31 E32 E33
E31 E33
RIF1
281 bp
204 bp
GAPDH
131 bp
GFP TadA-TadA*
P=2.36e-04
Inclusion (%)
GFP TadA-TadA*
e
GFP
3618
4101
70
TadA-TadA*
1988
2182
241
GADD45A
67,685,500
67,685,600
67,685,700
67,685,800
Genomic coordinate (chr1),"+" strand
GADD45A
E1 E2 E3
E1 E3
258 bp
156 bp
GAPDH
131 bp
GFP TadA-TadA*
P=1.37e-05
Inclusion (%)
GFP TadA-TadA*
f
GFP
80
12
2
TadA-TadA*
79
46
5
APEH
49,674,400
49,674,500
49,674,600
49,674,700
49,674,800
Genomic coordinate (chr3),"+" strand
APEH
E1 E2 E4
E1 E4
376 bp
245 bp
GAPDH
131 bp
GFP TadA-TadA*
P=5.16e-05
Inclusion (%)
GFP TadA-TadA*

Supplementary Figure 5. Validation of DAS events identified from RNA-seq data of HEK293T cells.

a) Sashimi plot and PCR validation of ADRM1 gene in GFP and BE3-treated cells. The alternative exon (Exon 4 (E4)) is marked in gray.

b) Sashimi plot and PCR validation of TCEAL9 gene in GFP and BE3-treated cells. The alternative exon (Exon 2 (E2)) is marked in gray.

c) Sashimi plot and PCR validation of PPP5C gene in GFP and BE3-treated cells. The alternative exon (Exon 5 (E5)) is marked in gray.

d) Sashimi plot and PCR validation of RIF1 gene in GFP and TadA-TadA*-treated cells. The alternative exon (Exon 32 (E32)) is marked in gray.

e) Sashimi plot and PCR validation of GADD45A gene in GFP and TadA-TadA*-treated cells. The alternative exon (Exon 2 (E2)) is marked in gray.

f) Sashimi plot and PCR validation of APEH gene in GFP and TadA-TadA*-treated cells. The alternative exon (Exon 2 (E2)) is marked in gray. Error bars represent S.D. for 3 independent experiments.

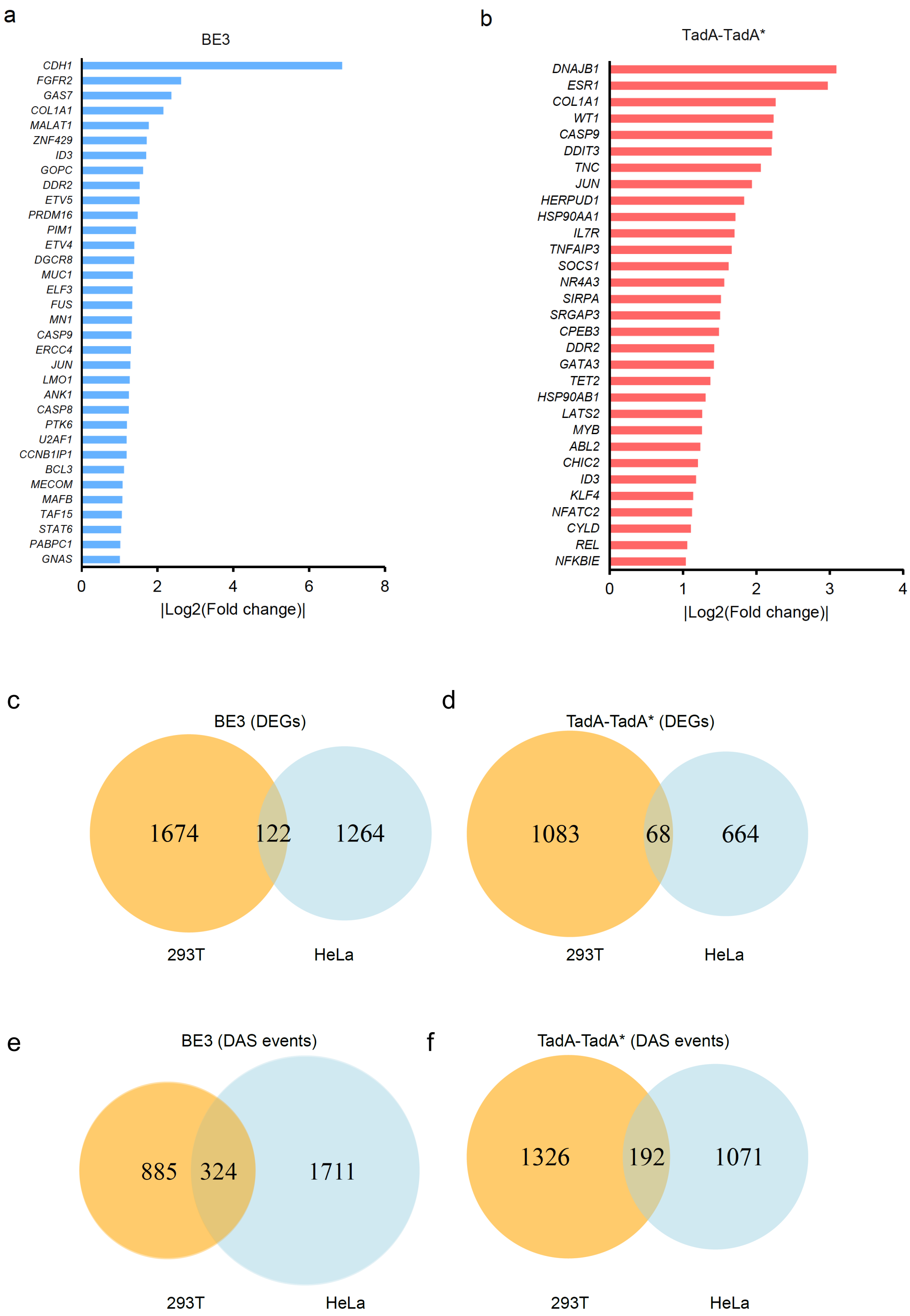

Supplementary Figure 6. Validation of DEGs identified from RNA-seq data of HeLa cells.
a) Cancer-related genes in BE3-induced DEGs of HeLa cells.
b) Cancer-related genes in TadA-TadA*-induced DEGs of HeLa cells.
c-f) Summary of DEG and DAS events between 293T and HeLa cells.

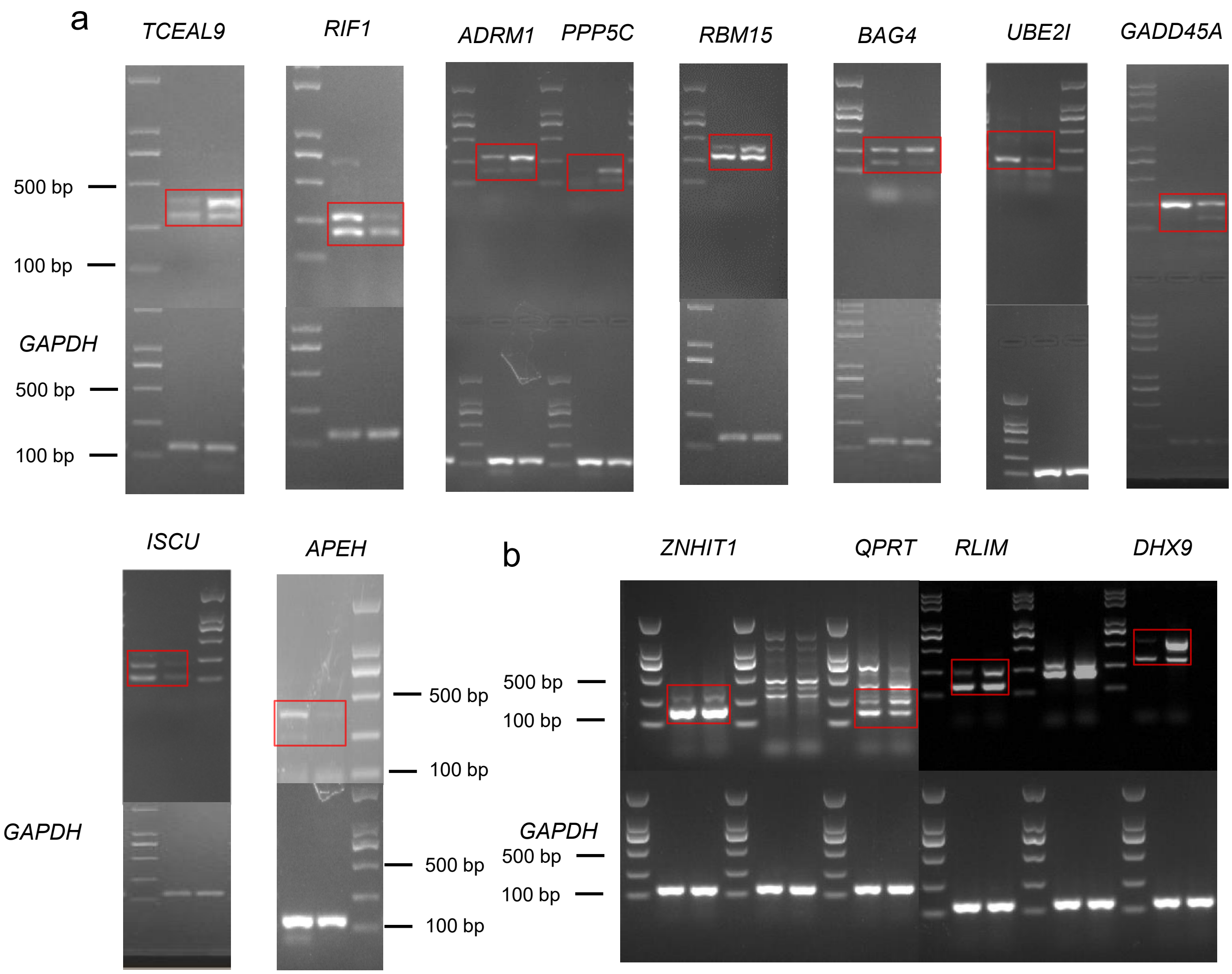

Supplementary Figure 7. Uncropped and unedited gel images of HEK293T cells and HeLa cells.

a) Uncropped and unedited gel images of HEK293T cells.

b) Uncropped and unedited gel images of HeLa cells.Size markers are as shown. The target bands are inside red boxs.

Supplementary Tables

Supplementary Table 1. The off-target RNA SNVs identified in CBEs

CBE groups	APOBEC1	BE3	BE3-site 3	BE3-RNF2
Editing sites	17826	12393	6530	8893
DEGs	1178	757	172	316
DEGs mapped to editing sites	60 (5.1%)	26 (3.4%)	9 (5.2%)	15 (4.7%)
DAS events	1779	568	1821	1612
DAS mapped to editing sites	81 (4.6%)	9 (1.6%)	30 (1.6%)	23 (1.4%)

Supplementary Table 2. The off-target RNA SNVs identified in CBEs

ABE groups	TadA-TadA*	ABE7.10	ABE7.10-site 1	ABE7.10-site 2
Editing sites	7501	3495	3506	6661
DEGs	1761	859	215	357
DEGs mapped to editing sites	196 (11.1%)	44 (5.1%)	19 (8.8%)	28 (7.8%)
DAS events	1949	1324	513	1115
DAS mapped to editing sites	86 (4.4%)	33 (2.5%)	11 (2.1%)	44 (3.9%)
