# Supplementary material for: Cytosine and adenine deaminase base-editors induce broad and nonspecific changes in gene expression and splicing
Source: Commun Biol. 2021 Jul 16;4:882. doi: 10.1038/s42003-021-02406-5 (PMC8285404; doi:10.1038/s42003-021-02406-5)
Supplement: Supplementary file 3 — Description of Additional Supplementary Files [file 42003_2021_2406_MOESM3_ESM.pdf]

Description of Additional Supplementary Files

Supplementary Data 1: DEGs identified in HEK293T cells transfected with APOBEC1, BE3, BE3-site 3, BE3-RNF2, TadA-TadA*, ABE7.10, ABE7.10-site 1, ABE7.10-site 2 compared with GFP samples.

Sheet1: DEGs of APOBEC1-transfected samples compared with GFP samples.

Sheet2: DEGs of BE3-transfected samples compared with GFP samples.

Sheet3: DEGs of BE3-site 3-transfected samples compared with GFP samples.

Sheet4: DEGs of BE3-RNF2-transfected samples compared with GFP samples.

Sheet5: DEGs of TadA-TadA*-transfected samples compared with GFP samples.

Sheet6: DEGs of ABE7.10-transfected samples compared with GFP samples.

Sheet7: DEGs of ABE7.10-site 1-transfected samples compared with GFP samples.

Sheet8: DEGs of ABE7.10-site 2-transfected samples compared with GFP samples.

Supplementary Data 2: GO Terms of BE3- or ABE7.10-transfected samples.

Sheet1: GO Terms of BE3-transfected samples.

Sheet2: GO Terms of ABE7.10-transfected samples

Supplementary Data 3: DEGs identified in HEK293T cells transfected with BE3$^{W90Y/R126E}$-site 3, BE3^{W90A}-site 3, BE3(hA3A)-site 3, BE3(hA3A^{R128A})-site 3, BE3(hA3A^{Y130F})-site 3, ABE7.10^{D53E}-site 1, ABE7.10^{F148A}-site 1, ABE7.10^{F148A}-site 2 compared with GFP samples.

Sheet1: DEGs of BE3$^{W90Y/R126E}$-site 3-transfected samples compared with GFP samples.

Sheet2: DEGs of BE3^{W90A}-site 3-transfected samples compared with GFP samples.

Sheet3: DEGs of BE3(hA3A)-site 3-transfected samples compared with GFP samples.

Sheet4: DEGs of BE3(hA3A^{R128A})-site 3-transfected samples compared with GFP samples.

Sheet5: DEGs of BE3(hA3A^{Y130F})-site 3-transfected samples compared with GFP samples.

Sheet6: DEGs of ABE7.10^{D53E}-site 1-transfected samples compared with GFP samples.

Sheet7: DEGs of ABE7.10^{F148A}-site 1-transfected samples compared with GFP samples.

Sheet8: DEGs of ABE7.10^{F148A}-site 2 -transfected samples compared with GFP samples.

Supplementary Data 4: DEGs identified in HEK293T cells transfected with ABEmax, miniABEmax, miniABEmax (K20A/R21A) or miniABEmax (V82G) and one of three different gRNAs (HEK site 2, ABE site 16 and a non-targeted control (NT)) compared with GFP samples. Sheet1: DEGs of ABEmax-HEK site 2-transfected samples compared with GFP samples.

Sheet2: DEGs of ABEmax-ABE site 16-transfected samples compared with GFP samples.

Sheet3: DEGs of ABEmax-NT-transfected samples compared with GFP samples.

Sheet4: DEGs of miniABEmax-HEK site 2-transfected samples compared with GFP samples.

Sheet5: DEGs of miniABEmax-ABE site 16-transfected samples compared with GFP samples.

Sheet6: DEGs of miniABEmax-NT-transfected samples compared with GFP samples.

Sheet7: DEGs of miniABEmax (K20A/R21A)-HEK site 2-transfected samples compared with GFP samples.

Sheet8: DEGs of miniABEmax (K20A/R21A)-ABE site 16-transfected samples compared with GFP samples.

Sheet9: DEGs of miniABEmax (K20A/R21A)-NT-transfected samples compared with GFP samples.

Sheet10: DEGs of miniABEmax (V82G)-HEK site 2-transfected samples compared with GFP samples. Sheet11: DEGs of miniABEmax (V82G)-ABE site 16-transfected samples compared with GFP samples.

Sheet12: DEGs of miniABEmax (V82G)-NT-transfected samples compared with GFP samples.

Supplementary Data 5: DEGs identified in HEK293T cells transfected with ABEmax, miniABEmax, miniABEmax (K20A/R21A) or miniABEmax (V82G) and one of three different

gRNAs (HEK site 2, ABE site 16 and a non-targeted control (NT)) compared with nCas9-NT samples.

Sheet1: DEGs of ABEmax-HEK site 2-transfected samples compared with nCas9-NT samples.

Sheet2: DEGs of ABEmax-ABE site 16-transfected samples compared with nCas9-NT samples.

Sheet3: DEGs of ABEmax-NT-transfected samples compared with nCas9-NT samples.

Sheet4: DEGs of miniABEmax-HEK site 2-transfected samples compared with nCas9-NT samples.

Sheet5: DEGs of miniABEmax-ABE site 16-transfected samples compared with nCas9-NT samples.

Sheet6: DEGs of miniABEmax-NT-transfected samples compared with nCas9-NT samples.

Sheet7: DEGs of miniABEmax (K20A/R21A)-HEK site 2-transfected samples compared with nCas9-NT samples.

Sheet8: DEGs of miniABEmax (K20A/R21A)-ABE site 16-transfected samples compared with nCas9-NT samples.

Sheet9: DEGs of miniABEmax (K20A/R21A)-NT-transfected samples compared with nCas9-NT samples.

Sheet10: DEGs of miniABEmax (V82G)-HEK site 2-transfected samples compared with nCas9-NT samples.

Sheet11: DEGs of miniABEmax (V82G)-ABE site 16-transfected samples compared with nCas9-NT samples.

Sheet12: DEGs of miniABEmax (V82G)-NT-transfected samples compared with nCas9-NT samples.

Supplementary Data 6: DEGs identified in HEK293T cells expressed WT BE3, SECURE-BE3 (R33A), SECURE-BE3 (R33A/K34A), hA3A-BE3, eA3A-BE3 and hAID-BE3 with co-expression of a gRNA targeting a site in the RNF2 gene compared with nCas9 samples.

Sheet1: DEGs of WT BE3-transfected samples compared with nCas9 samples.

Sheet2: DEGs of BE3(R33A)-transfected samples compared with nCas9 samples.

Sheet3: DEGs of BE3(R33A/K34A)-transfected samples compared with nCas9 samples.

Sheet4: DEGs of hA3A-BE3-transfected samples compared with nCas9 samples.

Sheet5: DEGs of eA3A-BE3-transfected samples compared with nCas9 samples.

Sheet6: DEGs of hAID-BE3-transfected samples compared with nCas9 samples.

Supplementary Data 7: DAS events identified in HEK293T cells transfected with APOBEC1, BE3, BE3-site 3, BE3-RNF2, TadA-TadA*, ABE7.10, ABE7.10-site 1, ABE7.10-site 2 compared with GFP samples.

Sheet1: DAS events of APOBEC1-transfected samples compared with GFP samples.

Sheet2: DAS events of BE3-transfected samples compared with GFP samples.

Sheet3: DAS events of BE3-site 3-transfected samples compared with GFP samples.

Sheet4: DAS events of BE3-RNF2-transfected samples compared with GFP samples.

Sheet5: DAS events of TadA-TadA*-transfected samples compared with GFP samples.

Sheet6: DAS events of ABE7.10-transfected samples compared with GFP samples.

Sheet7: DAS events of ABE7.10-site 1-transfected samples compared with GFP samples.

Sheet8: DAS events of ABE7.10-site 2-transfected samples compared with GFP samples.

Supplementary Data 8: DAS events identified in HEK293T cells transfected with BE3$_{W90Y/R126E}$-site 3, BE3^{W90A}-site 3, BE3(hA3A)-site 3, BE3(hA3A^{R128A})-site 3, BE3(hA3A^{Y130F})-site 3, ABE7.10^{D53E}-site 1, ABE7.10^{F148A}-site 1, ABE7.10^{F148A}-site 2 compared with GFP samples. Sheet1: DAS events of BE3$^{W90Y/R126E}$-site 3-transfected samples compared with GFP samples.

Sheet2: DAS events of BE3^{W90A}-site 3-transfected samples compared with GFP samples.

Sheet3: DAS events of BE3(hA3A)-site 3-transfected samples compared with GFP samples.

Sheet4: DAS events of BE3(hA3A^{R128A})-site 3-transfected samples compared with GFP samples.

Sheet5: DAS events of BE3(hA3A^{Y130F})-site 3-transfected samples compared with GFP samples.

Sheet6: DAS events of ABE7.10^{D53E}-site 1-transfected samples compared with GFP samples.

Sheet7: DAS events of ABE7.10^{F148A}-site 1-transfected samples compared with GFP samples.

Sheet8: DAS events of ABE7.10^{F148A}-site 2 -transfected samples compared with GFP samples.

Supplementary Data 9: DAS events identified in HEK293T cells transfected with ABEmax, miniABEmax, miniABEmax(K20A/R21A) or miniABEmax(V82G) and one of three different gRNAs (HEK site 2, ABE site 16 and a non-targeted control (NT)) compared with GFP samples. Sheet1: DAS events of ABEmax-HEK site 2-transfected samples compared with GFP samples. Sheet2: DAS events of ABEmax-ABE site 16-transfected samples compared with GFP samples. Sheet3: DAS events of ABEmax-NT-transfected samples compared with GFP samples.

Sheet4: DAS events of miniABEmax-HEK site 2-transfected samples compared with GFP samples.

Sheet5: DAS events of miniABEmax-ABE site 16-transfected samples compared with GFP samples.

Sheet6: DAS events of miniABEmax-NT-transfected samples compared with GFP samples.

Sheet7: DAS events of miniABEmax (K20A/R21A)-HEK site 2-transfected samples compared with GFP samples.

Sheet8: DAS events of miniABEmax (K20A/R21A)-ABE site 16-transfected samples compared with GFP samples.

Sheet9: DAS events of miniABEmax (K20A/R21A)-NT-transfected samples compared with GFP samples.

Sheet10: DAS events of miniABEmax (V82G)-HEK site 2-transfected samples compared with GFP samples.

Sheet11: DAS events of miniABEmax(V82G)-ABE site 16-transfected samples compared with GFP samples.

Sheet12: DAS events of miniABEmax(V82G)-NT-transfected samples compared with GFP samples.

Supplementary Data 10: DAS events identified in HEK293T cells transfected with ABEmax, miniABEmax, miniABEmax(K20A/R21A) or miniABEmax(V82G) and one of three different gRNAs (HEK site 2, ABE site 16 and a non-targeted control (NT)) compared with nCas9-NT samples.

Sheet1: DAS events of ABEmax-HEK site 2-transfected samples compared with nCas9-NT samples.

Sheet2: DAS events of ABEmax-ABE site 16-transfected samples compared with nCas9-NT samples.

Sheet3: DAS events of ABEmax-NT-transfected samples compared with nCas9-NT samples.

Sheet4: DAS events of miniABEmax-HEK site 2-transfected samples compared with nCas9-NT samples.

Sheet5: DAS events of miniABEmax-ABE site 16-transfected samples compared with nCas9-NT samples.

Sheet6: DAS events of miniABEmax-NT-transfected samples compared with nCas9-NT samples.

Sheet7: DAS events of miniABEmax(K20A/R21A)-HEK site 2-transfected samples compared with nCas9-NT samples.

Sheet8: DAS events of miniABEmax(K20A/R21A)-ABE site 16-transfected samples compared with nCas9-NT samples.

Sheet9: DAS events of miniABEmax(K20A/R21A)-NT-transfected samples compared with nCas9-NT samples.

Sheet10: DAS events of miniABEmax(V82G)-HEK site 2-transfected samples compared with nCas9-NT samples.

Sheet11: DAS events of miniABEmax(V82G)-ABE site 16-transfected samples compared with nCas9-NT samples.

Sheet12: DAS events of miniABEmax(V82G)-NT-transfected samples compared with nCas9-NT

samples.

Supplementary Data 11: DAS events identified in HEK293T cells expressed WT BE3, SECURE-BE3(R33A), SECURE-BE3(R33A/K34A), hA3A-BE3, eA3A-BE3 and hAID-BE3 with co-expression of a gRNA targeting a site in the RNF2 gene compared with nCas9 samples.

Sheet1: DAS events of WT BE3-transfected samples compared with nCas9 samples.

Sheet2: DAS events of BE3(R33A)-transfected samples compared with nCas9 samples.

Sheet3: DAS events of BE3(R33A/K34A)-transfected samples compared with nCas9 samples.

Sheet4: DAS events of hA3A-BE3-transfected samples compared with nCas9 samples.

Sheet5: DAS events of eA3A-BE3-transfected samples compared with nCas9 samples.

Sheet6: DAS events of hAID-BE3-transfected samples compared with nCas9 samples.

Supplementary Data 12: DEGs and DAS events identified in HEK293T cells transfected with BE3, and TadA-TadA* compared with GFP samples.

Sheet1: DEGs of BE3-transfected samples compared with GFP samples.

Sheet2: DEGs of TadA-TadA*-transfected samples compared with GFP samples.

Sheet3: DAS events of BE3-transfected samples compared with GFP samples.

Sheet4: DAS events of TadA-TadA*-transfected samples compared with GFP samples.

Supplementary Data 13: DEGs and DAS events identified in HeLa cells transfected with BE3, and TadA-TadA* compared with GFP samples.

Sheet1: DEGs of BE3-transfected HeLa cells compared with GFP samples.

Sheet2: DEGs of TadA-TadA*-transfected HeLa cells compared with GFP samples.

Sheet3: DAS events of BE3-transfected HeLa cells compared with GFP samples.

Sheet4: DAS events of TadA-TadA*-transfected HeLa cells compared with GFP samples.

Supplementary Data 14: DEGs and DAS events identified in HEK293T cells that expressed TadA–TadA* or APOBEC1 in gradient concentration (1.5 µg, 2.5 µg, 3.5 µg) compared with GFP samples.

Sheet1: DEGs of APOBEC1-transfected cells (1.5 jig) compared with GFP samples.

Sheet2: DEGs of APOBEC1-transfected cells (2.5 jig) compared with GFP samples.

Sheet3: DEGs of APOBEC1-transfected cells (3.5 jig) compared with GFP samples.

Sheet4: DEGs of TadA-TadA*-transfected cells (1.5 jig) compared with GFP samples.

Sheet5: DEGs of TadA-TadA*-transfected cells (2.5 jig) compared with GFP samples.

Sheet6: DEGs of TadA-TadA*-transfected cells (3.5 jig) compared with GFP samples.

Sheet7: DAS events of APOBEC1-transfected cells (1.5 jig) compared with GFP samples.

Sheet8: DAS events of APOBEC1-transfected cells (2.5 jig) compared with GFP samples.

Sheet9: DAS events of APOBEC1-transfected cells (3.5 jig) compared with GFP samples.

Sheet10: DAS events of TadA-TadA*transfected cells (1.5 jig) compared with GFP samples.

Sheet11: DAS events of TadA-TadA*-transfected cells (2.5 jig) compared with GFP samples.

Sheet12: DAS events of TadA-TadA*-transfected cells (3.5 jig) compared with GFP samples.

Supplementary Data 15: Sequences for primers used in this study.
