# Supplementary material for: Cytosine and adenine deaminase base-editors induce broad and nonspecific changes in gene expression and splicing
Source: Commun Biol. 2021 Jul 16;4:882. doi: 10.1038/s42003-021-02406-5 (PMC8285404; doi:10.1038/s42003-021-02406-5)
Supplement: Supplementary file 19 — Reporting Summary [file 42003_2021_2406_MOESM19_ESM.pdf]

nature research

Corresponding author(s): Wenjie Shu

Last updated by author(s): Jun 10, 2021

Reporting Summary

Nature Research wishes to improve the reproducibility of the work that we publish. This form provides structure for consistency and transparency in reporting. For further information on Nature Research policies, see our Editorial Policies and the Editorial Policy Checklist.

Statistics

For all statistical analyses, confirm that the following items are present in the figure legend, table legend, main text, or Methods section.

n/a	Confirmed	
☐	☒	The exact sample size (n) for each experimental group/condition, given as a discrete number and unit of measurement
☐	☒	A statement on whether measurements were taken from distinct samples or whether the same sample was measured repeatedly
☐	☒	The statistical test(s) used AND whether they are one- or two-sided<br>*Only common tests should be described solely by name; describe more complex techniques in the Methods section.*
☐	☒	A description of all covariates tested
☒	☐	A description of any assumptions or corrections, such as tests of normality and adjustment for multiple comparisons
☐	☒	A full description of the statistical parameters including central tendency (e.g. means) or other basic estimates (e.g. regression coefficient) AND variation (e.g. standard deviation) or associated estimates of uncertainty (e.g. confidence intervals)
☐	☒	For null hypothesis testing, the test statistic (e.g. F, t, r) with confidence intervals, effect sizes, degrees of freedom and P value noted<br>*Give P values as exact values whenever suitable.*
☒	☐	For Bayesian analysis, information on the choice of priors and Markov chain Monte Carlo settings
☒	☐	For hierarchical and complex designs, identification of the appropriate level for tests and full reporting of outcomes
☐	☒	Estimates of effect sizes (e.g. Cohen's d, Pearson's r), indicating how they were calculated

Our web collection on statistics for biologists contains articles on many of the points above.

Software and code

Policy information about availability of computer code

Data collection	GENCODE v30
Data analysis	FastQC v0.11.8, TrimGalore v0.6.1, STAR v2.5.0a, HTSeq v0.10.0.

For manuscripts utilizing custom algorithms or software that are central to the research but not yet described in published literature, software must be made available to editors and reviewers. We strongly encourage code deposition in a community repository (e.g. GitHub). See the Nature Research guidelines for submitting code & software for further information.

Data

Policy information about availability of data

All manuscripts must include a data availability statement. This statement should provide the following information, where applicable:

- Accession codes, unique identifiers, or web links for publicly available datasets
- A list of figures that have associated raw data
- A description of any restrictions on data availability

All sequencing data have been deposited under GEO with number GSE158693. The source data underlying Figure 4, Figure 5 and Figure 6.

Field-specific reporting

Life sciences study design

All studies must disclose on these points even when the disclosure is negative.

Sample size	Sample size was based on empirical data from pilot experiments.
Data exclusions	No data were excluded from the analyses.
Replication	Experimental findings were replicated successfully and data are presented as mean ± s.e.m.
Randomization	Experimental groups were defined by experimental conditions. Within an experimental condition, the allocation was random.
Blinding	The investigators were blinded to group allocation during data collection and analysis. All experimental procedures and quantification of results were done by two independent researchers.

Reporting for specific materials, systems and methods

We require information from authors about some types of materials, experimental systems and methods used in many studies. Here, indicate whether each material, system or method listed is relevant to your study. If you are not sure if a list item applies to your research, read the appropriate section before selecting a response.

Materials & experimental systems

n/a	Involved in the study	
☒	☐	Antibodies
☐	☒	Eukaryotic cell lines
☒	☐	Palaeontology and archaeology
☒	☐	Animals and other organisms
☒	☐	Human research participants
☒	☐	Clinical data
☒	☐	Dual use research of concern

Methods

n/a	Involved in the study	
☒	☐	ChIP-seq
☒	☐	Flow cytometry
☒	☐	MRI-based neuroimaging

Eukaryotic cell lines

Policy information about cell lines

Cell line source(s)	HEK293T (ATCC CRL-3216) and HeLa (ATCC CCL-2) were obtained from the American Type Culture Collection (ATCC).
Authentication	We purchased the cell lines from the American Type Culture Collection (ATCC).
Mycoplasma contamination	The cell lines were tested for mycoplasma contamination using MycoAlert Mycoplasma Detection Kit (Lonza, #LT07-418).
Commonly misidentified lines (See ICLAC register)	No commonly misidentified cell line is used.
